# Development of sodium acetate trihydrate-ethylene glycol composite phase change materials with enhanced thermophysical properties for thermal comfort and therapeutic applications

**DOI:** 10.1038/s41598-017-05310-3

**Published:** 2017-07-12

**Authors:** Rohitash Kumar, Sumita Vyas, Ravindra Kumar, Ambesh Dixit

**Affiliations:** 1 Heat Management Group, Defence Laboratory Jodhpur, Rajasthan, 342011 India; 2 Department of Physics & Centre for Energy, Indian Institute of Technology Jodhpur, Rajasthan, 342011 India

## Abstract

The heat packs using phase change materials (PCMs) are designed for possible applications such as body comfort and medical applications under adverse situations. The development and performance of such heat packs rely on thermophysical properties of PCMs such as latent heat, suitable heat releasing temperature, degree of supercooling, effective heat releasing time, crystallite size, stability against spontaneous nucleation in metastable supercooled liquid state and thermal stability during heating and cooling cycles. Such PCMs are rare and the available PCMs do not exhibit such properties simultaneously to meet the desired requirements. The present work reports a facile approach for the design and development of ethylene glycol (EG) and aqueous sodium acetate trihydrate (SAT) based composite phase change materials, showing these properties simultaneously. The addition of 2–3 wt% EG in aqueous SAT enhances the softness of SAT crystallites, its degree of supercooling and most importantly the effective heat releasing time by ~10% with respect to aqueous SAT material. In addition, the maximum heat releasing temperature of aqueous SAT has been tailored from 56.5 °C to 55 °C, 54.9 °C, 53.5 °C, 51.8 °C and 43.2 °C using 2%, 3%, 5%, 7% and 10 wt% EG respectively, making the aqueous SAT-EG composite PCMs suitable for desired thermal applications.

## Introduction

The extreme low ambient temperature at high altitude regions adversely affects the health of inhabitants and may cause frostbite, hypothermia etc. Physiological alterations in the vital organs like brain, digestive and respiratory system may also occur due to the prolonged exposure to the very low temperature. Hence, it is necessary to provide the desired thermal conditions using external heating source and insulating mediums to reduce thermal losses in such situations for mitigating these problems. At present, hot water bottles, electric heating pads/blankets etc. are being used as external heat source to provide comfort in such adverse conditions^1^. The insulating mediums such as winter clothing and blankets provide relief by reducing the heat transfer rate between body and environment. However, these do not provide any heating and thus may not be suitable for longer exposure. Hot water bottles provide initial heating at higher temperature, which reduces rapidly to the lower temperatures due to the low thermal energy density of water. Thus, such heating source may provide thermal solution for a very short duration. Temperature of electric based heating devices, such as pads, blankets etc. is controlled externally using the thermostat and may lead to the causalities such as skin burn in the case of its failure. In addition, such devices are not suitable for field applications, where electrical sources may not be available. In countries like India, inhabitants use Kangaries as heat source to keep body warm during outdoor activities in high altitude regions such as Kashmir. The use of Kangaries is harmful for the health because of inhaling the toxic gases e.g. carbon mono/dioxide, released while burning the coal. Simultaneously, there are possibilities of skin burning incidents and cutaneous cancer also^2^. Bukharies are also being used for indoor heating applications, but these are also harmful to health as burning kerosene/wood/coal is being used as a fuel, which produces harmful gases^3^. Sometimes, death causalities have also been observed, when Bukharies are used for heating in closed rooms. The closed environment may lack oxygen inside the room and thus, inhaling carbon mono-oxide with respiration while sleeping may lead to such causalities.

Thus, alternative renewable, reusable and cost effective heating sources are required to provide the thermal comfort under such conditions. These alternative heating sources should be self-sufficient to store available thermal energy such as solar energy, waste heat etc. at high temperature (~60 °C), retain it at lower ambient temperature (subzero) and release the heat later at the time of requirement. The development of such heating sources will rely on the materials having high thermal energy storage density and suitable heat releasing temperature with a possibility of desired time delay between thermal energy storage and supply. Phase change materials (PCMs) can serve such purpose because of their high latent heat of fusion at desired temperatures. In addition, the latent heat thermal energy storage materials are more efficient than sensible heat storage materials such as water, due to their high-energy storage density and storage/release of heat in narrow temperature range, near the phase transition temperature of PCMs ^4–7^. These superior properties of PCMs make them attractive to develop thermal management devices/systems for regulating human body temperature, reduction of indoor temperature fluctuations inside a building and seasonal solar heat energy storage. In addition to such applications, thermal phase change materials with suitable thermophysical properties may also be used for peak electricity load shifting, storage of blood, medicines, food and beverages etc. at desired safe temperatures, thermal management of electronic devices, solar water heater, solar cookers and thermal energy storage systems (TESs) for solar power plants etc^8–12^.

Phase change materials such as hydrated salts exhibit large degree of supercooling, which is not desirable for general heat storage applications such as building heating/cooling. However, this property can be utilized for long-term storage of thermal energy at low ambient temperature and release it later at the time of demand by initiating the crystallization in the metastable supercooled liquid PCMs^13, 14^. Among such PCMs, sodium acetate trihydrate (SAT) has attracted attention because of its high latent heat of fusion ~250 kJ/kg, suitable melting temperature ~58 °C, large supercooling range, better thermal stability during charge/discharge cycles and most important, the low price^15–20^. Such thermal properties and low cost of SAT make it suitable for reusable PCM thermal devices such as heat packs, heating of shelters and seasonal solar energy storage applications. These reusable PCM heat packs are being used widely for body warming to get rid of cold during field operations and therapeutic applications such as treatment of hypothermia, muscle aches, and frostbite. These heat packs are made of poly vinyl chloride (PVC), low density polyethylene (LDPE), high density polyethylene (HDPE) etc. packaging materials and contain aqueous solution of sodium acetate trihydrate PCM and metallic triggering device^21^. In these heat packs, PCM remains in metastable supercooled liquid state, far below its solidification temperature. This property helps in storing the thermal energy as the latent heat of PCM, even at low ambient temperature (~0 °C) for very long time. Thus, PCM heat packs can store heat/waste heat/solar thermal energy at the time of availability and release it later at the time of requirement by initiating the solidification in metastable supercooled liquid state of PCM using triggering device. The rate of solidification in these heat packs is very fast due to the metastable supercooled liquid state of PCM and is usually completed in few seconds^22–24^.

**Figure 1:**
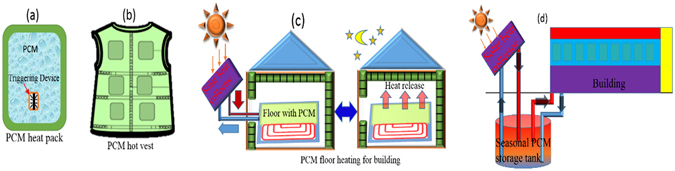
Schematic representation for use of sodium acetate trihydrate PCM in (**a**) reusable PCM heat pack, (**b**) PCM hot vest, (**c**) PCM floor heating for buildings and (**d**) seasonal solar thermal energy storage and building heating.

In spite of such thermo-physical properties of SAT and potential applications in heating devices as explained schematically in Fig. 1, there are few challenges associated with SAT based PCM heat packs such as hard, lumped, big and sharp edges of SAT crystallites, unintentional nucleation in metastable supercooled liquid PCM at low ambient temperature (~0 °C), low heat retention time due to the fast crystal growth during solidification from metastable supercooled liquid state.

SAT based heat packs provide heating at ~57 °C, which limits its usage for different applications such as instant heating of tissues after frostbite where heating at ~40 °C is required. The hard and lumped salt crystallites limit the flexibility of thermal devices, which hinders the shape adaptability of SAT based heat pack. In addition, the sharp SAT crystallite edges are prone to puncture/damage the packaging material, and also causes discomfort to the human body during therapeutic applications. SAT and SAT based binary eutectic systems have been studied extensively for latent heat thermal energy storage applications^25–31^. However, less efforts are made to mitigate such shortcomings of SAT for its possible applications. Considering the same, we have used ethylene glycol (EG) as an additive in aqueous SAT, and investigated thermophysical and microstructural properties of SAT and SAT-EG composite materials. We observed that ethylene glycol (EG) helps in reducing aqueous SAT crystallite size and increases the effective heat retention time about 10% with respect to aqueous SAT phase change material. We will discuss the observed thermophysical properties of investigated materials using structural, microstructural, thermal characterizations and the microscopic origin of enhanced thermal properties of aqueous SAT-EG composite phase change materials.

## Results

### X-ray Diffraction Analysis

**Figure 2:**
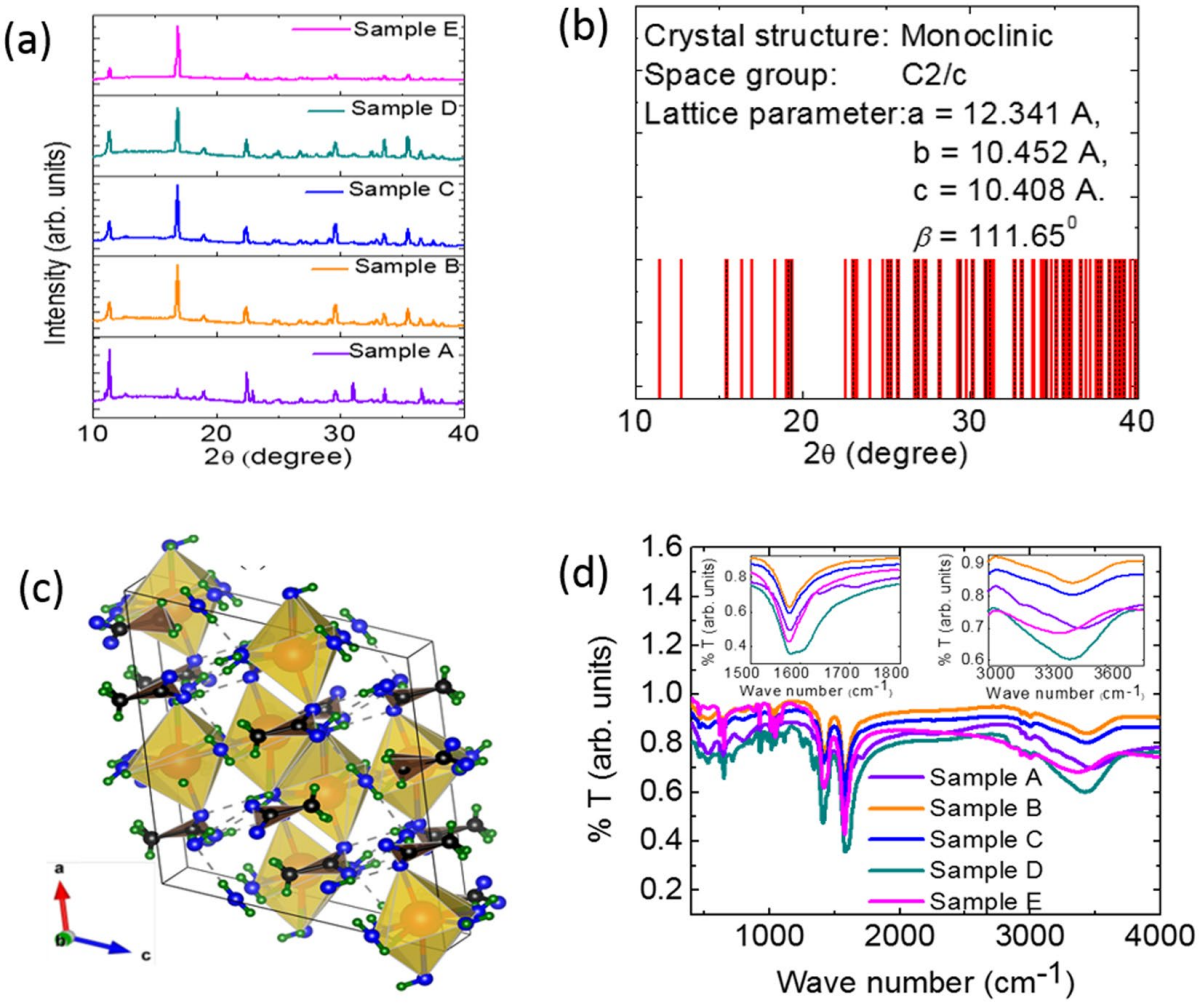
Measured powder X-ray diffractographs for samples A, C, D and E, (**b**) calculated X-ray diffraction pattern with inset showing lattice parameters, (**c**) crystal structure of SAT crystal with Orange large sphere: Na atom; blue smaller circle: Oxygen atom, green smaller circle: hydrogen atom, black circle: carbon atom and (**d**) FTIR spectrographs of sample A, C, D and E.

The crystal structure of aqueous SAT (94 wt% sodium acetate trihydrate + 6 wt% deionized water) named as sample A hereafter, and EG modified aqueous SAT samples by 2, 3, 5, and 7 wt% EG are named sample B, C, D and E, respectively hereafter in text. The crystallographic information for these samples are investigated using powder X-ray diffraction (pXRD) measurements in 10°–40° 2Θ range and results are summarized in Fig. 2(a).

The XRD pattern of aqueous SAT is in good agreement with the reported monoclinic crystallographic structure with space group C2/c^32^. The XRD pattern is used to calculate the lattice structural parameters and agree with the reported literature values^32^. The theoretical XRD pattern has been calculated for monoclinic structure (space group C2/c) using the estimated lattice parameters. The generated pattern is summarized in Fig. 2(b) and observed that generated diffraction pattern exhibits only those hkl diffraction patterns for which h + k = 2n; (h0l) with h = 2n, l = 2n and (0k0) with k = 2n (n is an integer). These diffraction patterns substantiate the observed C2/c space group for aqueous SAT. The crystallographic structure of aqueous SAT has been generated using these structural parameters and shown in Fig. 2(c) with polyhedron around sodium atoms. These edge shared polyhedrons form long chains, may be responsible for long needle shaped crystals as observed in microscopic studies and discussed later. The XRD pattern for EG modified aqueous SAT samples (sample B, C, D and E) are identical to that of the sample A. However, the relative intensity of these diffraction patterns has reduced with increase in EG wt% ratio. The reduced intensity is attributed to the geometrical effects, where smaller crystals have resulted in the out of phase diffracted radiation, and thus relatively lower intensity. This has been substantiated with our microscopic observations, discussed later. The XRD measurement was not possible for sample F (Sample A with 10 wt% EG) due to its semi-liquidous characteristics at room temperature (~25 °C).

### Fourier Transform Infrared Spectroscopic Analysis

The room temperature Fourier Transform Infrared (FTIR) spectroscopic measurements are carried out on these samples. The respective spectrographs are shown in Fig. 2(d). FTIR spectrographs for -OH bending vibrations (1500–1800 cm^−1^ wavenumber) and -OH stretching vibrations (3000–3800 cm^−1^ wavenumber) of these samples are shown in insets of Fig. 2(d). These insets suggest that -OH bending and stretching vibrational frequencies shift towards higher and lower frequencies with increasing wt% of EG in aqueous SAT-EG composite materials respectively. The shifting of vibrational frequencies in FTIR vibrational spectra suggests that there are hydrogen bond interactions between H atoms of water and hydroxyl oxygen atoms of ethylene glycol^33^. The observed interaction between H (aqueous SAT) – OH (EG) may assist in reducing the melting temperature and increasing the supercooling temperature of aqueous SAT-EG composites against the aqueous SAT phase change material.

### Microstructural Analysis

**Figure 3:**
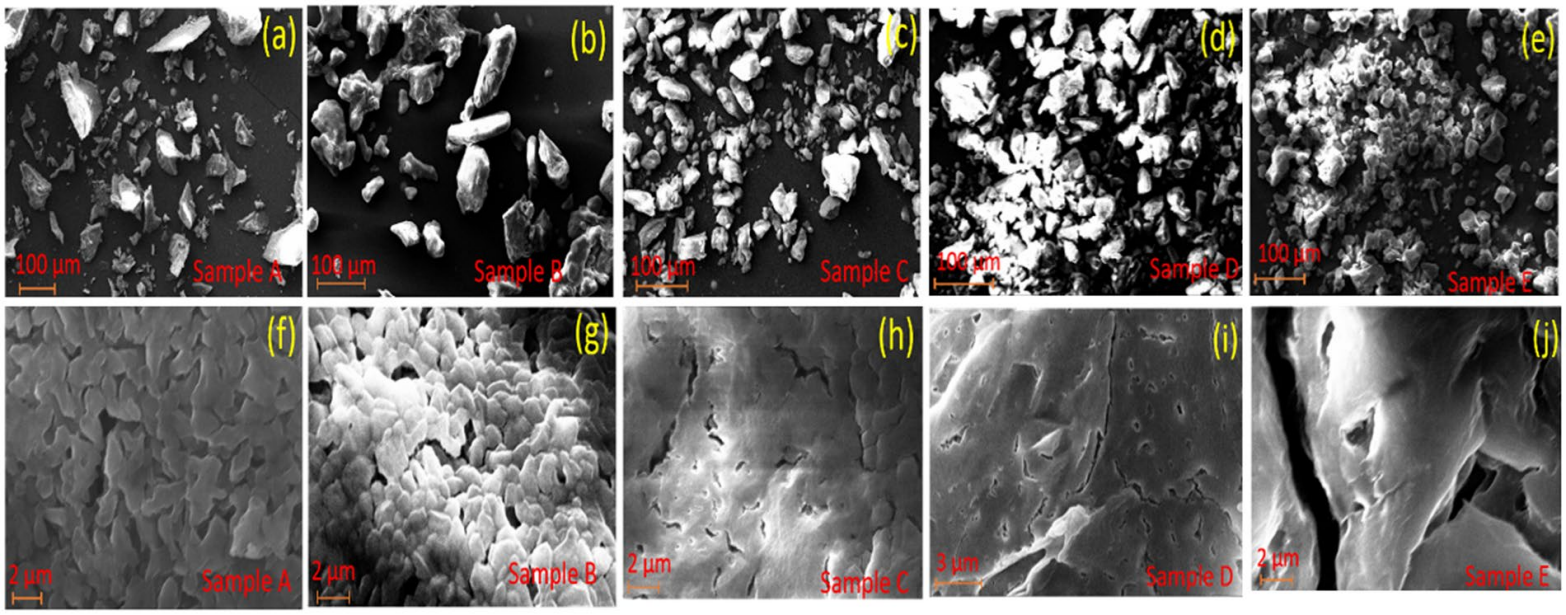
Scanning electron micrographs with 500 X (top panel) and 15000 X (bottom panel) magnifications for sample A, B, C, D and E.

The microstructural properties of samples A, B, C, D and E are investigated using scanning electron microscope (SEM) and respective micrographs are summarized in Fig. 3(a–j) at 500× and 15 k × magnifications respectively.

Figure 3(a) explains the sharp and big SAT crystallites, which are substantiated by the pXRD observations with relatively large diffraction intensity (Fig. 2(a)), as explained above. The sharp and big crystals reduce the shape adaptability and flexibility of heat pack as per requirements. The SEM micrographs shown in Fig. 3(b–e) explain the reduction in size and edge sharpness of aqueous SAT crystallites with increasing wt% of EG. These results are in good agreement with pXRD results, Fig. 2(a), where the reduced intensity and enhanced FWHM are observed for samples B, C, D, and E with respect to sample A. This reduction in the diffraction intensity suggests that long range ordered crystallite phase (Fig. 3(a)), has converted into short range ordered microstructures (Fig. 3(b–e)). The SEM micrograph of sample A (Fig. 3(f)), explains the tightly packed crystallites, which are strong and difficult to break into smaller crystallites. Figure 3(g–j) explains the insertion of liquid EG into aqueous SAT crystallites during crystal growth, thus, weakening the crystals and assisting the reduction of crystallite size. The increasing EG wt% in aqueous SAT has led to the enhanced insertion of EG and thus, reducing the crystallite size into micro/Nano crystallites, as explained in Fig. 3(g–j).

### Thermal Properties Analysis

#### Temperature-history measurements

**Figure 4:**
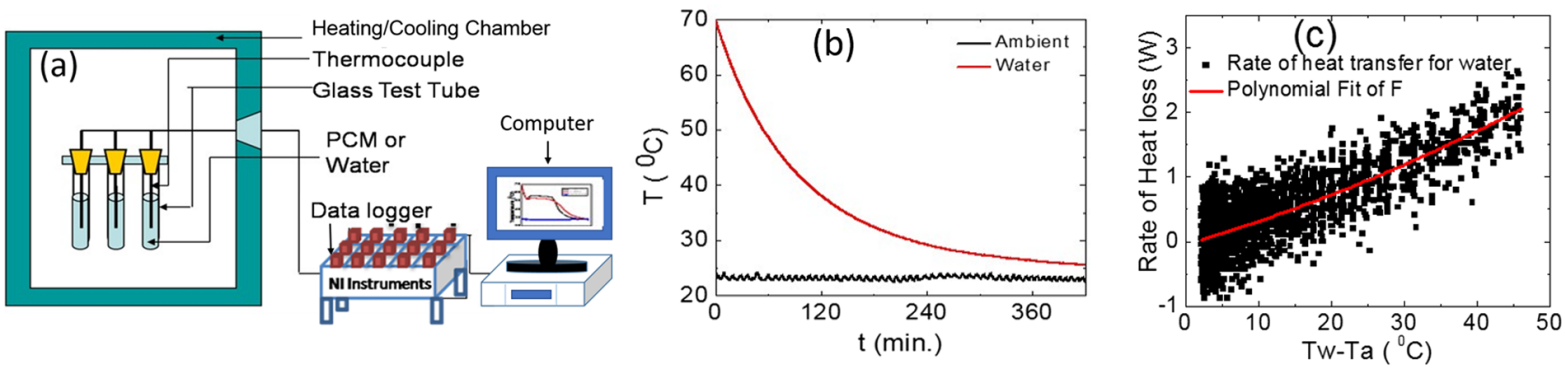
(**a**) Schematic of semi-automated T-history measuring test setup, (**b**) T-history curve for DI water, (**c**) calculated heat loss from water test tube vs. temperature difference between ambient and DI water temperature.

We carried out T-history measurements to measure the thermal response of samples under investigation using an in-house built temperature history (T-history) set-up^34^. The schematic of experimental T-history set-up is shown in Fig. 4(a). The system consists of a controlled heating/cooling insulated chamber of size 60 cm (L) × 32 cm (W) × 30 cm (H), 500 W capacity electric heater for heating samples, 600 W capacity refrigeration system for cooling samples, variable air circulating fan to regulate the temperature uniformity inside the chamber, k-type thermocouples integrated with data logger and a computer system for recording the temperature versus time data. The temperature inside T-history chamber can be varied in the range of 0–100 °C as per requirement.

The heating and cooling measurements on samples can be carried out in this T-history set-up and repeated sets of measurements can be carried out with better accuracy as the measurements are carried out without disturbing experimental setup^35^. There are different approaches to generate enthalpy vs. temperature curves for phase change materials using T-history measurements^36–45^. We have used Sandanes approach to analyze the T-history data and evaluate the thermophysical properties of investigated PCMs^36^. The deionized (DI) water is used as a reference sample. All the PCM samples and deionized water are collected in glass test tubes of size 15 mm diameter and 200 mm height. The weight of each sample has been kept constant (50 g) for T-history measurements. The physical properties of glass test tubes, all PCMs and reference samples, used for these measurements, are listed in Table 1. These samples are heated up to 80 °C inside the chamber as explained in Fig. 4(a) for carrying out temperature vs. time (T-history) measurements and data is recorded at every 10 second time interval. The sample containers are kept inside 150 mm diameter and 280 mm height cylindrical expanded polypropylene (EPP) thermal insulations. This effective thermal insulation is important to realize the low heating and cooling rates with minimal temperature gradients within the sample. This is important to achieve a low Biot number (<0.1) to use lump capacitance method for calculating the thermophysical properties of these materials with better accuracy^46, 47^.

**Table 1:** Physical properties of test tubes, PCMs and DI water used for experiments.

Sample name	Sample details	mass of test tube (g)	mass of sample material (g)	Specific heat of test tube (kJ.kg^−1^. K^−1^)
Reference	Deionized water	28.057	50	0.84
A	94 wt% SAT + 6 wt% DI water	28.123	50	0.84
B	Sample A + 2 wt% EG	28.427	50	0.84
C	Sample A + 3 wt% EG	28.235	50	0.84
D	Sample A + 5 wt% EG	28.326	50	0.84
E	Sample A + 7 wt% EG	28.148	50	0.84
F	Sample A+ 10 wt% EG	28.445	50	0.84

Initially, a water sample is heated inside T-history chamber up to 80 °C, (above the melting temperature of sample A ~57 °C) and then cooled to the ambient temperature (23 ± 0.5 °C), (below the solidification temperature of sample A) to record T-history data for water and ambient temperature. The recorded T-history data are plotted in Fig. 4(a) for deionized water during cooling from 70 °C to the ambient temperature. Small periodic oscillations have been observed in ambient (chamber) temperature data because of the lag in compressor and controller feedback process. This small variation in temperature has no significant impact on PCM T-history measurements, as the temperature difference of successive temperatures measured at every 10 sec intervals has been considered for evaluating thermophysical properties. These recorded T-history data are used for calculating the rate of heat loss from deionized water sample in 70–25 °C cooling temperature range, using the following equation:

1 $${\dot{q}}_{w,i}=({m}_{w}{c}_{p,w}+{m}_{t}{c}_{p,t})\frac{{\rm{\Delta }}{T}_{w,i}}{{\rm{\Delta }}{t}_{i}}$$

Where ${\dot{q}}_{w,i}$ is the rate of heat loss from water test tube, *m*_*w*_ mass of water, *c*_*p,w*_ constant pressure specific heat capacity of water (4.18 kJ.kg^−1^.K^−1^), *m*_*t*_ mass of glass test tube, *c*_*p,t*_ constant pressure specific heat capacity of test tube (0.84 kJ.kg^−1^.K^−1^), ${\rm{\Delta }}{T}_{w,i}={T}_{w,i+1}-{T}_{w,i}$ temperature difference between two consecutive measurements of water and ${\rm{\Delta }}{t}_{i}={t}_{i+1}-{t}_{i}$ is time interval between two consecutive measurements, which is 10 second for these measurements^36^. The calculated rate of heat loss from deionized water sample versus temperature difference between deionized water and ambient (T_w_ − T_amb_) is plotted in Fig. 4(c). A second order polynomial has been fitted to these heat loss data, as shown by red solid line and used to estimate the heat loss coefficients *k*_0_, *k*_1_ and *k*_2_, as described in the following equation:

2 $${\dot{q}}_{(Tw-Tamb)}={k}_{0}+\,{k}_{1}({T}_{w}-{T}_{amb})+{k}_{2}{({T}_{w}-{T}_{amb})}^{2}$$

The calculated *k*_0_, *k*_1_ and *k*_2_ coefficient values are −0.0425 W, 0.03252 W/°C and 2.8363 × 10 − 4 W/°C^2^ respectively.

**Figure 5:**
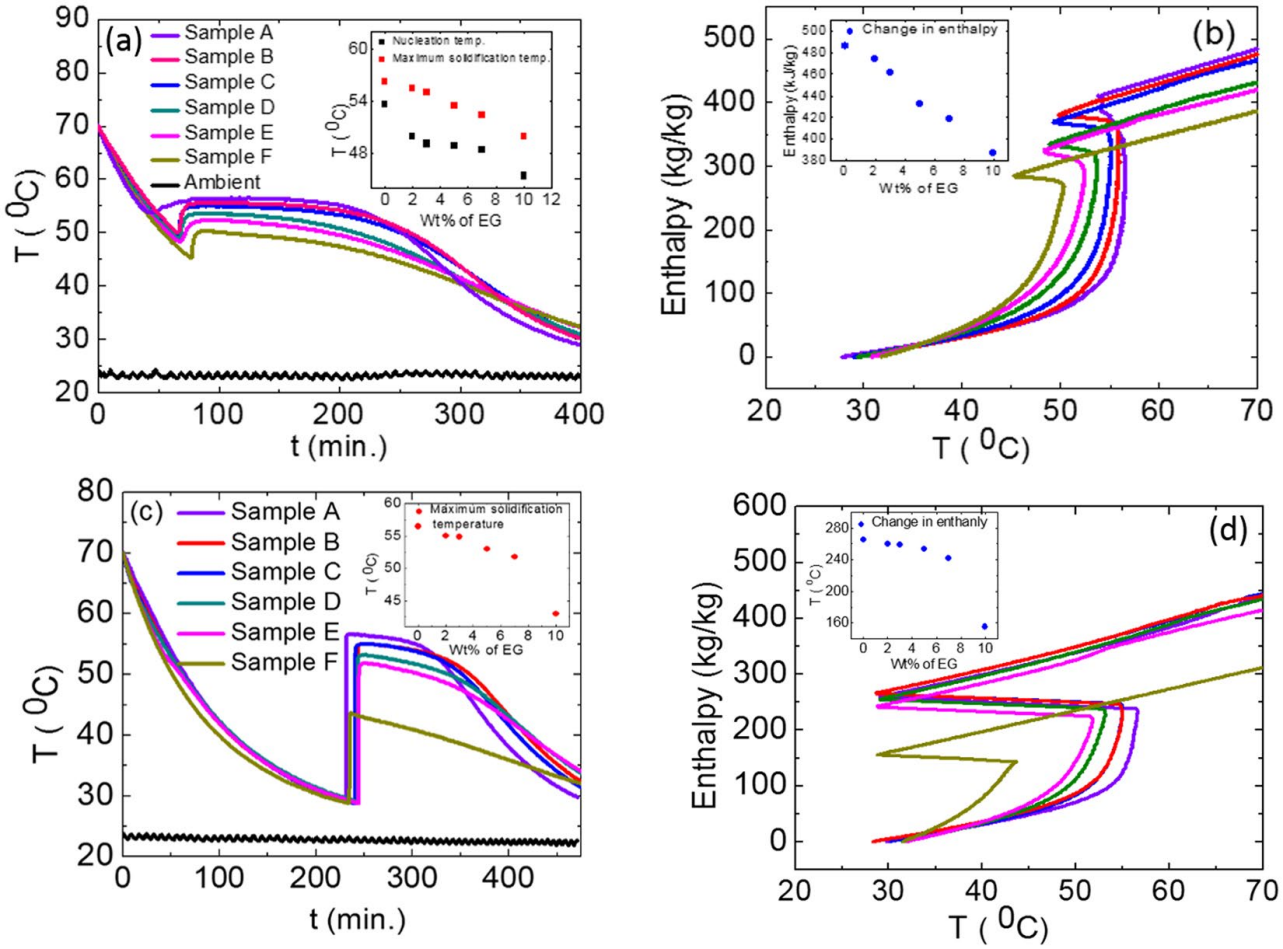
(**a**) T-history graphs for samples A, B, C, D, E and F nucleated at 60 °C and ambient temperature ~23 °C, (**b**) enthalpy vs. temperature curves corresponding to graph 5(**a**), (**c**) T-history graphs for samples A, B, C, D, E and F nucleated at ~30 °C and ambient temperature ~23 °C, (**d**) enthalpy vs. temperature curve corresponding to graph 5(**c**).

The similar T-history measurements are carried out for A, B, C, D, E and F samples. The sample weights, insulation, positioning of k-type thermocouples and ambient temperature conditions are kept identical during the measurements as explained above under T-history measurements section. The cooling T-history data has been recorded from 70 °C to ~30 °C with external nucleation in liquid samples at ~60 °C using solid SAT powder. The measured data are summarized in Fig. 5(a), with the respective ambient temperature.

The temperature of sample A is exhibiting undercooling up to 53.6 °C and after that nucleation started in this supercooled liquid, which led to instant rise in temperature up to 56.6 °C. Samples B, C, D, E and F have shown enhanced supercooling temperature up to 49.9 °C, 49.1 °C, 48.9 °C, 48.4 °C, and 45.4 °C respectively, as explained in the inset of Fig. 5(a). These observations suggest the enhancement in the degree of supercooling for aqueous SAT-EG composite samples with increasing wt% of EG. Thus, aqueous SAT-EG composite samples may exhibit better stability against spontaneous nucleation in metastable supercooled liquid state with respect to aqueous SAT sample A. In addition, the maximum temperature achieved during solidification (heat releasing temperature) of sample A shows decrease with increasing wt% of EG and the maximum reduction has been observed for 10 wt% EG in aqueous SAT composite sample, as explained in the inset of Fig. 5(a). These studies provide an avenue where variation of EG can be used as a parameter to tailor the heat releasing temperature and degree of supercooling of aqueous SAT PCM, required for different applications. The first order derivatives of T-history measurements, not shown here, are used to find out completion of liquid-solid phase transformation temperature more accurately^40^. We observed that liquid-solid phase transformation completion temperature decreases with increasing wt% fraction of EG i.e. the liquid to solid phase transformation temperature range has increased with increasing EG fraction. The samples B and C take ~10% excess time to cool from 70 °C to 40 °C as compared to the sample A for the same temperature range. This suggests that 2–3 wt% EG modified samples (sample B and C) may provide thermal energy for longer time durations, thus more suitable for heat pack applications. However, samples with higher wt% EG in sample A (samples D, E and F) showed nearly the same time as that sample A for cooling from 70 °C to 40 °C temperature range. This is attributed to the reduction in respective enthalpies with increase in wt% EG, as observed in Fig. 5(b).

These temperature versus time measurements in conjunction with the calculated heat loss coefficients *k*_0_, *k*_1_ and *k*_2_ for deionized water are used to calculate the combined rate of heat loss from PCM and test tubes containing PCM samples, using following equation:

3 $${\dot{q}}_{PCM+testtube,i}={k}_{0}+{k}_{1}({T}_{PCM,i}-{T}_{amb,i})+{k}_{2}{({T}_{PCM,i}-{T}_{amb,i})}^{2}\,$$

and the rate of heat loss from PCM has been calculated using following equation:

4 $${\dot{q}}_{PCM,i}={\dot{q}}_{PCM+testtube,i}+({m}_{t}{C}_{pt})\frac{({T}_{PCM,i+1}-{T}_{PCM,i})}{({t}_{i+1}-{t}_{i})}$$

where ${\dot{q}}_{PCM+testtube,i}$ and ${\dot{q}}_{PCM,i}$ are the rate of heat loss from PCM and test tube, the rate of heat loss from PCM at i^th^ time interval, *m*_*t*_ mass of test tube, *c*_*p,t*_ constant pressure specific heat capacity of test tube (0.84 J/g°C), ${T}_{PCM,i+1}-{T}_{PCM,i}$ temperature difference between two consecutive measurements of PCM and *t*_*i*+1_ − *t*_*i*_ is time interval between two consecutive measurements, which is 10 seconds for these measurements. The calculated rate of heat loss from PCM, equation (4), is used to estimate the change in enthalpy (Δ*H*_*PCM,i*_) of PCM for i^th^ time interval using following equation:

5 $${\rm{\Delta }}{H}_{PCM,i}({T}_{PCM,i})=\frac{{\dot{q}}_{PCM,i}{\rm{\Delta }}{t}_{i}}{{m}_{PCM}}$$

The calculated Δ*H*_*PCM,i*_ are summed to calculate the cumulative enthalpy of PCM samples with respect to the temperature. The calculated cumulative enthalpy versus temperature graphs, for Fig. 5(a), are plotted in Fig. 5(b). The cumulative enthalpy of sample A, B, C, D and E and F at temperature 70 °C, nucleated at 60 °C is plotted as an inset in Fig. 5(b), suggesting that the enthalpy of sample A decreases with increasing wt% of EG in SAT. These results are consistent with the expected reduction because of reduced fraction of aqueous SAT in aqueous SAT-EG composite phase change materials.

Additional T-history measurements are carried out for these samples, which are supercooled up to 30 °C and nucleated at this temperature using SAT fine powder. The measured T-history data are plotted in Fig. 5(c). A sharp increase in temperature has been observed for all these samples after external nucleation. This rise in temperature is a consequence of latent heat release while solidification. This temperature rise has been summarized in the inset of Fig. 5(c), suggesting decrease in temperature rise with increasing wt% of EG. This is consistent with that of observed, while nucleating at 60 °C (inset Fig. 5(a)). Inset in Fig. 5(c) suggests that the heat releasing temperature can be tailored from 57 to 43 °C by simply varying wt% of EG up to 10% in aqueous SAT. The respective enthalpy versus temperature data are calculated as explained above and the results are plotted in Fig. 5(d). The enthalpies of these samples at 30 °C against wt% EG are plotted as an inset in Fig. 5(d). The enthalpies of these samples (nucleated at 30 °C) are lower as comparted to that of samples, nucleated at 60 °C. The lower enthalpy values for samples, nucleated at 30 °C is mainly due to loss of thermal energy in the form of specific heat of liquid during PCM cooling from 70 to 30 °C.

### Differential Scanning Calorimetric Measurements

**Figure 6:**
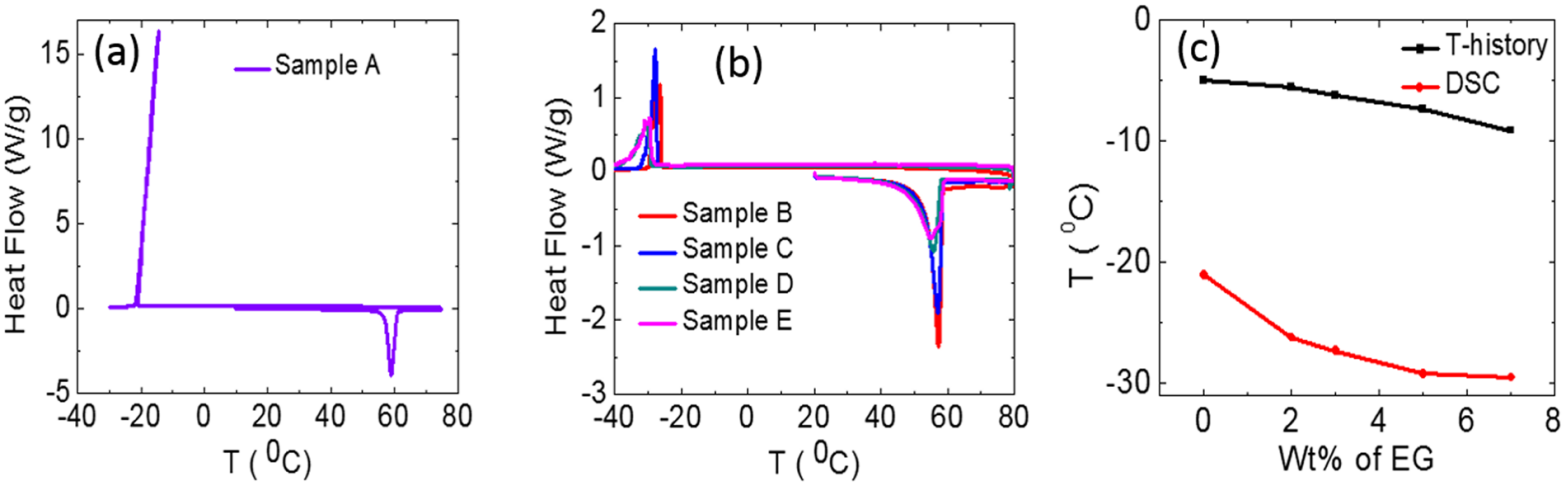
Differential scanning calorimetric (DSC) thermograph for sample A (**a**); samples B, C, D, and E (**b**) and supercooling temperature for these samples, measured using DSC and T-history methods (**c**).

Further, thermophysical properties of these phase change materials are substantiated by Differential Scanning Calorimetric (DSC) measurements using DSC TA Q10 (TA Instruments USA make). The measuring sample is hermetically sealed in an aluminum pan and empty aluminum pan is used simultaneously as a reference sample. The instrument is calibrated using Indium reference material before carrying out DSC measurements on these samples. The weights of sample A, B, C, D and E used for DSC measurements are 5.19 mg, 5.12, 5.86, 6.27 and 6.08 mg respectively. The measurements are carried out at identical heating and cooling rate of 2 °C/min under inert environment using continuous nitrogen gas purging at 50 ml/min flow rate. The melting and solidification of PCM samples are identified as endothermic (down) and exothermic peaks (up) in DSC thermographs shown in Fig. 6(a,b).

Sample A exhibited sharp (narrow temperature window) endothermic and exothermic DSC peaks, as shown in Fig. 6(a). In contrast to sample A, EG modified composite PCM (samples B, C, D and E) showed wider endothermic and exothermic DSC peaks, Fig. 6(b). The narrow temperature range of exothermic peak for sample A suggests high rate of heat release in narrow temperature window as compared to EG modified composite PCM samples. The onset and endset temperatures and latent heat of fusion for these PCM samples are estimated using DSC thermographs, Fig. 6(a and b), and results are summarized in Table 2. The observations are consistent with T-history observations, suggesting that melting/solidification temperature decreases and melting/solidification temperature range increases with increasing wt% of EG in aqueous SAT. The self-nucleating temperature of aqueous SAT decreases with increasing wt% of EG, suggesting enhancement in degree of supercooling and thermal stability of metastable supercooled liquid against spontaneous nucleation.

**Table 2:** Melting and solidification temperatures of SAT and SAT + EG composite PCMs.

Sample Name	Onset temperature for melting peak of DSC (°C)	Endset temperature for melting peak of DSC (°C)	Onset temperature for solidification peak of DSC (°C)	Endset temperature for solidification peak of DSC (°C)	Latent heat of fusion (kJ.kg^−1^)
A	57.6	60.1	−21.0	−21.68	256.7
B	54.3	58.2	−26.2	−29.2	251.54
C	53.5	57.8	−27.3	−31.8	249.31
D	49.5	56.8	−29.2	−37.0	236.35
E	47.8	55.8	−29.5	−38.1	222.82

The measured degree of supercooling for samples A, B, C, D and E using both DSC and T-history methods are plotted in Fig. 6(c). This suggests that the degree of supercooling has enhanced for EG modified composite PCM samples. The values of self-nucleating temperatures for all samples measured with DSC are lower, as compared to that from T-history measurements. The difference between these results are due to the variation in the mass of samples, used for these measurements. In DSC, a very small amount (few milligrams) of the sample is being used for the measurements, which reduces the probability of nucleation as compared to the voluminous sample (few tens of grams), used in T-history measurements. Hence, T-history measurement values may be more reliable as compared to that of DSC measurements, for any real application, where usually large quantities of samples are used.

### Fabrication and Evaluation of PCM Heat Packs

**Figure 7:**
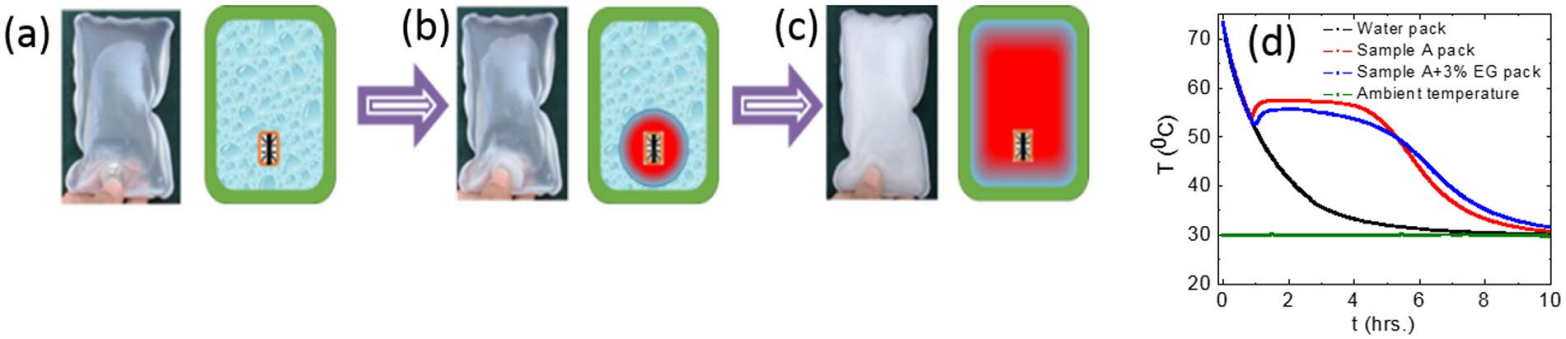
Schematic and pictorial representation of heat releasing process from PCM heat packs: (**a**) flexing triggering device to start nucleation in supercooled liquid PCM for its solidification at t = 0 second, (**b**) growth of solidification at t = 5 seconds, (**c**) complete solidification of supercooled liquid PCM at t = 55 seconds and (**d**) T-history curves for heat packs consisting of water, sample A and C.

Sample C (aqueous SAT with 3 wt% EG) is used for fabrication of PCM heat packs because of its superior thermophysical properties such as heat releasing temperature, latent heat of fusion, degree of supercooling, effective heat releasing time, small crystallites. Three heat packs, containing 300 g water, sample A and sample C, are fabricated to compare the thermal performance. Polyvinyl chloride (PVC) has been used as a packaging material for storing PCM and triggering device. The stainless-steel triggering device, consisting surface imperfections, is kept inside PCM packs to release heat at the time of requirement. The actual photograph and schematic of such fabricated heat pack (110 mm × 180 mm) and solidification mechanism in PCM heat packs is explained in Fig. 7(a–c). The triggering device is flexed to start nucleation for solidification of PCM in heat packs, Fig. 7(a). This solidification propagates in all the three directions, Fig. 7(b) and takes around one minute for complete solidification of PCM, Fig. 7(c). The thermal energy equivalent to the PCM latent heat is released during this process.

The heat packs are kept inside an insulated box (size 150 mm × 220 mm × 10 mm) made of 20 mm thick EPP material, to ensure thermal equilibrium across the heat pack during T-history measurements. This insulated box is kept inside T-history heating-cooling chamber and heat packs are heated up to 80 °C, followed by cooling up to ambient temperature ~30 °C. The thermal response of these heat packs is recorded from 75 to 30 °C at every 10 second time interval. The PCM inside the heat packs is nucleated at 60 °C above its melting temperature. The measured T-history responses of these three packs are plotted in Fig. 7(d). Water, sample A and C based heat packs took about 2.24, 6.4 and 7.03 hours respectively to cool from 75 to 40 °C temperature. Thus, extended heating time for heat packs containing sample A and C is about 186% and 214% more as compared to the water heat pack. Further, an enhancement of ~10% additional heat release time has been observed for sample C based heat pack with respect to sample A based heat pack.

## Discussion

**Figure 8:**
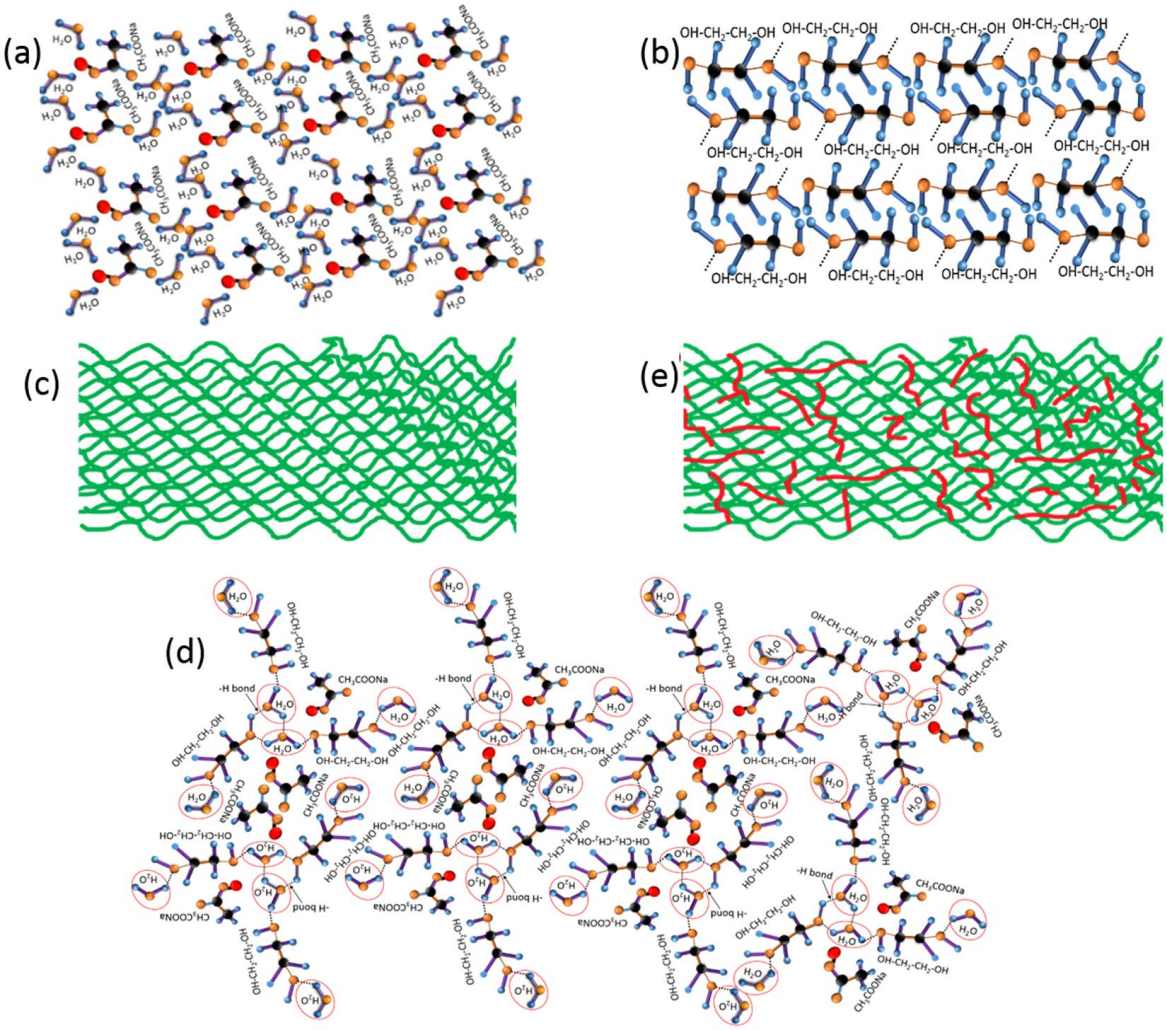
Schematic representation of SAT molecules (**a**), EG molecules (**b**), macroscopic arrangement of SAT crystals (**c**), hydrogen bond interactions between water H atoms of SAT and hydroxyl oxygens of EG (**d**), and macroscopic arrangement for insertion of EG as laminar vesicles (red lines) in SAT matrix (**e**). The blue, black, yellow and red spheres represent the H, C, O and Na atoms respectively. The hydrogen bond between hydrogen atom of water and hydroxyl atom of EG is represented with doted black lines.

The proposed interactions between sodium acetate trihydrate and ethylene glycol at atomic level is shown schematically in Fig. 8, based on the molecular interactions, inferred from FTIR measurements.

The schematic molecular structure of SAT and EG are shown in Fig. 8(a and b) respectively. The metastable supercooled liquid SAT solidifies in large and lumped continuous crystallites, as shown schematically in Fig. 8(c). Such large lumped crystallites are not suitable for thermal heat pack applications. This can be mitigated by using the physical mixing of EG in SAT matrix. Here, EG is dispersed homogeneously in SAT matrix because of weak hydrogen bond interaction between SAT water hydrogen atoms and EG hydroxyl oxygen atoms, as observed and inferred from FTIR spectra of SAT-EG composite samples. The interaction mechanism is explained schematically in Fig. 8(d). The uniform dispersion of EG assists in the adsorption of liquid EG layers on the growing SAT crystal faces. Thus, these liquid EG layers inhibits the growth of large and lumped SAT crystallites. This liquid EG forms the laminar vesicles, as observed in microscopic studies, Fig. 3. The process of laminar vesicles formation in SAT is explained schematically in Fig. 8(d). These laminar vesicles of liquid EG in SAT crystals are responsible for weakening the SAT crystallites and thus, reducing the continuous growth of SAT crystallites. Further, this inculcated liquid EG will assist in softening, by breaking crystallites with external stimuli causing shear forces between EG separated SAT planes, as shown schematically in Fig. 8(e). The hydrogen bonding between ethylene glycol and H_2_O molecules of SAT may disturbs the hydrogen bonding between SAT and H_2_O molecules. It may be the responsible for enhancement of activation energy required to form nuclei with critical radius and enhances the degree of supercooling and thermal stability of SAT-EG metastable supercooled liquid PCM against spontaneous nucleation compared to SAT.

In summary, novel ethylene glycol and aqueous sodium acetate trihydrate composite phase change materials with enhanced thermophysical properties have been designed and developed for different thermal applications. Thermophysical properties of aqueous SAT PCM, such as melting/solidification temperatures, degree of supercooling, heat retention/release time, have been tailored and optimized by varying EG weight fraction in aqueous SAT-EG composite phase change materials for specific applications. The optimal ~10% enhancement in heat retention time has been observed for 3 wt% EG modified aqueous SAT PCM, without affecting other thermophysical properties significantly. The thermal stability of metastable supercooled liquid SAT against spontaneous nucleation has enhanced significantly for aqueous SAT-EG composite PCMs. In addition, the insertion of liquid EG into SAT crystallites helps in controlling the SAT crystallites size by hindering the growth of large and lumped SAT crystallites. The thermal response of developed aqueous SAT- EG composite PCMs has shown promising thermophysical properties for possible applications such as therapeutic/body warming, building heating under adverse conditions and seasonal solar thermal energy storage. These studies suggest that aqueous SAT-EG composite PCM may improve the flexibility and physical stability of PCM heat packs for the desired applications.

## Materials and Methods

Sodium acetate trihydrate and ethylene glycol (EG) grade “excel R” are purchased from Qualigens Fine Chemicals Pvt. Ltd. Mumbai, India and used without any further purification.

### Preparation of aqueous SAT-EG composites

Aqueous SAT-EG composite samples are prepared by simple physical mixing process. The materials are weighed by a digital micro balance model CX65S (the accuracy of balance is ±0.01 mg, make Citizen, USA). The different aqueous SAT-EG composite samples are prepared by physical mixing of different wt% of EG in aqueous sodium acetate trihydrate (94% SAT + 6% DI water) using magnetic stirrer for 20 minutes at 70 °C. The six samples, sample A (94 wt% SAT + 6 wt% deionized H_2_O), sample B (sample A + 2 wt% EG), sample C (sample A + 3 wt% EG), sample D (sample A + 5 wt% EG), sample E (sample A + 7 wt% EG) and sample F (sample A + 10 wt% EG) are prepared. Additional deionized water in pristine SAT is added to enhance its thermal stability during heating/cooling cycles. The effect of EG on solidification temperature, maximum temperature during solidification, degree of supercooling, heat release time, stability of metastable supercooled liquid and softness of aqueous SAT have been investigated to develop aqueous SAT– EG composite systems as a phase change material for possible heat pack applications.

### Thermophysical characterization

The structural properties of aqueous SAT and aqueous SAT-EG composite samples are carried out using Bruker D8 Advance X-ray diffractometer using copper K_α_ radiation ((λ = 1.5406Å). The XRD measurements are recorded in the range of 10 to 40° with step size 0.02°. The microstructural properties of these samples are investigated using Carl Zeiss EVO 18 Scanning Electron Microscope (SEM). The vibrational modes of these materials are identified using Bruker vertex 70 v Fourier Transform Infrared (FTIR) spectroscopy system in the range of 4000–400 cm^−1^. An in-house fabricated semi-automated temperature-history (temperature versus time) system, as explained in Fig. 4(a), is used for thermal response measurements of these composite samples. K-type thermocouples (0.5 mm diameter) connected with National Instruments (NI-USA) data logger are used for collecting temperature versus time (T-history) measurements at every 10 second time interval. Differential scanning calorimeter (DSC) Q10 is used to measure latent heat of fusion, melting temperature of samples under investigation and validation of T-history results.
